# Effectiveness of pilot trial of family meal support prior inpatient discharge

**DOI:** 10.1186/2050-2974-2-S1-P14

**Published:** 2014-11-24

**Authors:** Mei Yee Chan, Helen Kelly

**Affiliations:** 1 The North Western Mental Health Eating Disorder Program at The Royal Melbourne Hospital, Melbourne, Australia

## 

The North Western Mental Health Eating Disorder Program at The Royal Melbourne Hospital provides treatment for adults 18 years and over since 1994. The eating disorder program provides an in-patient and day-patient treatment program and comprehensive out-patient services.

Family members have been an effective and vital support for clients with eating disorders in their treatment and recovery in our day patient program. This pilot trial is to explore the family dynamics and confidence of family members in supporting in-patient clients with anorexia nervosa during a meal. Family members would be empowered with evidence based knowledge and strategies to support their loved ones through a meal prior to discharge as part of the discharge planning.

The theoretical framework for this trial is based on a modification of the Family-Based Therapy (FBT). In several controlled studies, it has proven that Family-Based Treatment to be successful in an out-patient setting especially for adolescent in helping individual in weight restoration.

